# The genus *Rumex* (Polygonaceae): an ethnobotanical, phytochemical and pharmacological review

**DOI:** 10.1007/s13659-022-00346-z

**Published:** 2022-06-16

**Authors:** Jing-Juan Li, Yong-Xiang Li, Na Li, Hong-Tao Zhu, Dong Wang, Ying-Jun Zhang

**Affiliations:** 1 State Key Laboratory of Phytochemistry and Plant Resources in West China, Kunming Institute of Botany, Chinese Academy of Sciences, Kunming, 650201 People’s Republic of China; 2 University of Chinese Academy of Sciences, Beijing, 100049 People’s Republic of China; 3 Yunnan Key Laboratory of Natural Medicinal Chemistry, Kunming Institute of Botany, Chinese Academy of Sciences, Kunming, 650201 People’s Republic of China

**Keywords:** Polygonaceae, *Rumex* L., Anthraquinones, Phenolics, Pharmacological properties

## Abstract

*Rumex* L., a genus in Polygonaceae family with about 200 species, is growing widely around the world. Some *Rumex* species, called "sorrel" or "dock", have been used as food application and treatment of skin diseases and hemostasis after trauma by the local people of its growing areas for centuries. To date, 29 *Rumex* species have been studied to contain about 268 substances, including anthraquinones, flavonoids, naphthalenes, stilbenes, diterpene alkaloids, terpenes, lignans, and tannins. Crude extract of *Rumex* spp. and the pure isolates displayed various bioactivities, such as antibacterial, anti-inflammatory, antitumor, antioxidant, cardiovascular protection and antiaging activities. *Rumex* species have important potential to become a clinical medicinal source in future. This review covers research articles from 1900 to 2022, fetched from SciFinder, Web of Science, ResearchGate, CNKI and Google Scholar, using “*Rumex*” as a search term ("all fields") with no specific time frame set for the search. Thirty-five *Rumex* species were selected and summarized on their geographical distribution, edible parts, traditional uses, chemical research and pharmacological properties.

## Introduction

*Rumex* L., the second largest genus in the family Polygonaceae, with more than 200 species, is mainly distributed in the northern temperate zone [1]. It is mostly perennial herbs with sturdy roots, paniculate inflorescences, and triangular fruits that are enveloped in the enlarged inner perianth. The name "*Rumex*" originated from the Greek word–"dart" or "spear", alluding to the shape of leaves [2]. The other explanation from Rome–"rums" alludes to the function that the leaves could be sucked to alleviate thirst [3]. *R. acetosa*, a typical vegetable and medicinal plant, whose name 'acetosa' originated from the Latin word "acetum", described the taste of the plant as vinegar. Currently, many oxalic acids have been reported from *Rumex*, verifying its sour tastes [4].

*Rumex* species have had a valued place as global folk medicine, e.g., in Southern Africa, America, India, China, and Turkey. The earliest medicinal record of *Rumex* spp. in China was in "Shennong's Herbal Classic", in which *Rumex* was recorded for the treatment of headed, scabies, fever, and gynecological diseases. Roots of *Rumex*, also called dock root, have been reported for its therapeutic capacity of bacterial infections, inflammatory, tumor and cardiovascular diseases [5, 6]. Recently, pharmacological study showed that *Rumex* species displayed apparent antibacterial and antifungal effects [7], and were employed in the management of skin scabies and inflammation [8, 9]. The processed *Rumex* exhibited different chemical profiles and bioactivities [10, 11]. Leaves, flowers and seeds of some *Rumex* plants are edible as vegetables, while in some regions, the *Rumex* plants are regarded as noxious weeds because oxalic acid makes them difficult to be digested [12].

To date, 268 components from 29 *Rumex* species have been reported. Anthraquinones, flavonoids, tannins, stilbenes, naphthalenes, diterpene alkaloids, terpenes, and lignans were as the main chemical components, with a broad spectrum of pharmacological activities, such as anti-inflammatory, antioxidant, antibacteria, antitumor, and antidiabetic activities [13–17]. In addition to important role of *Rumex* in the traditional applications, researchers also regard *Rumex* as a potential effective medicine of many diseases. This article has reviewed a comprehensive knowledge on the distribution, traditional uses, chemistry and bioactivity progress of *Rumex*, and their therapeutic applications and utilizations were provided.

## Geographical distributions, local names, parts used and traditional uses

The genus *Rumex* with more than 200 species, is distributed widely in the world and has been used traditionally in many regions, e.g., Asia, America, Europe and other continents. Many of them known as "sorrel" or "dock" have a long history of food application and medicinal uses for the treatment of skin diseases, and hemostasis after trauma by the local people of its growing areas. For example, *R*. *acetosa* is commonly used medicinally for diuretics around the world [4]. *R*. *maritimus* and *R*. *nepalensis*, used as laxatives, have long-term medicinal applications in India as substitutes for *Rheum palmatum* (Polygonaceae), which is usually used to regulate the whole digestive system. Moreover, Indians have also recorded nine *Rumex* plants as astringent agents, including *R*. *acetosa*, *R*. *acetosella*, *R*. *crispus*, *R*. *dentatus*, *R*. *hastatus*, *R*. *maritimus*, *R*. *nepalensis*, *R*. *scutatus*, and *R*. *vesicarius* [18]. All seven species included *R. acetosa*, *R. trisetifer*, *R. patientia*, *R. crispus*, *R. japonicus*, *R. dentatus* and *R. nepalensis*, called "jinbuhuan", have been used for hemostasis remediation in China [19]. *R*. *thyrsiflorus*, rich in nutrition, has been used as food by Europeans in history and as folk medicine due to its obvious anti-inflammatory activity [20]. *R*. *lunaria* has been used to treat diabetes by Canarian medicine [16]. The leaves of more than 14 *Rumex* spp., such as *R. acetosa*, *R*. *hastatus*, *R*. *thyrsiflorus*, *R*. *aquaticus*, *R*. *crispus*, *R*. *gmelini*, *R*. *patientia*, *R*. *vesicarius*, *R*. *ecklonianus*, *R*. *abyssinicus*, *R*. *confertus*, *R*. *hymenosepalus*, *R*. *alpinus* and *R*. *sanguineus* (Table 1) could be eaten freshly or cooked as vegetables in the folk of many places [5, 6]. In Table 1, the geographical distributions, local names, parts used and traditional uses of 35 *Rumex* species are summarized.

**Table 1:** Traditional uses of *Rumex* plants

No	Species	Local names	Country	Parts used	Traditional uses	Ref
R1	*Rumex acetosa* L	Sorrel, garden sorrel, common dock, broad-leaved sorrel, English sorrel, sheep’s sorrel, red sorrel, sour weed, field sorrel	South Africa, North America, Europe, Yemen, Czech Repubilc, Korea, Britain, Ireland, China, Hungary, Romania and Bulgaria	Leaf, flower, whole plant, fruit, root and seed	Gastrointestinal disorders (constipation, cramping, diarrhea, tenesmus), antiscorbutic, hemostasis, dermatological, tumors, cramping, sore throats, warts, dysentery, gonorrhea, ulcer, scabies, kidney diseases (diuretic), fever, worm, abscesses. Seed: astringent	[4, 18, 19, 57, 135, 192, 198]
R2	*R. hastatus* D. Don	Heartwing sorrel, hastate-leaved dock, sour dock, khatimal	China, India, Nepal, Bhutan, Pakistan and Afghanistan	Leaf, flower, seed, root, whole plant, anile part and contemporary tuber	Astringent, sexually transmitted diseases (AIDs), constipation, tonic agent, diuretic, rheumatism, dermatological, piles, bleeding of the lungs, cough, headache, fever, blood pressure, abdominal pain, sore throat, tonsillitis diseases, worm, wounds	[18, 58, 191, 195]
R3	*R. thyrsiflorus* Fingerh	Compact dock, thyrse sorrel	China, Kazakhstan and Russia, and Europe	Leaf	For food	[59, 198]
R4	*R. aquaticus* L	Red dock, western dock	China, Japan, Kazakhstan, Russia and Europe	Leaf	Disinfection, constipation, fever, diarrhea, stomach problems, edema, jaundice	[60, 201]
R5	*R. Chalepensis* Mill		Asia, Middle East, Morocco and Africa	–	–	[40, 61, 202]
R6	*R. crispus* L	Curled dock, curly dock, yellow dock, narrow-leaf dock	Asia, Europe, North America, Northern Africa, Colombia and India	Leaf, root, stem, seed	Antidysentery, hemostasis, ulcers, cough. Root: laxative, astringent, skin eruptions, skin diseases, scrofula, scurvy, intermittent fevers, congested liver and jaundice. Seed: astringent	[18, 19, 62, 195, 197]
R7	*R. dentatus* L	Toothed dock	Asia, Middle East and Southeast Europe and Pakistan	Whole plant	Cutaneous disorders, stomach problems. Plant: astringent, hemostasis	[18, 19, 28, 63, 195]
R8	*R. gmelini* Turcz. ex Ledeb		China, Japan, North Korea, Russia, Mongolia and Siberia	Leaf	Tumor, bacterial infection	[31, 64]
R9	*R. japonicus* Houtt		China, Japan, North Korea and Russia	Whole plant	Hemostasis, fever, constipation	[19, 65, 199]
R10	*R. maritimus* L	Golden dock	Bangladesh, India, North Africa and America	Leaf, root and seed	Leaf and root: laxative; externally applied to burns. Seed: aphrodisiac	[18, 66]
R11	*R. nepalensis* Spreng		Asia, Europe and Africa, Ethiopia, Nepal, Pakistan and India	Leaf, root and whole plant	Hemostasis, stomach problems, itch, astringent, paralysis, tonsillitis, ascariasis, uterine bleeding, as an abortifacient, joint pain. Leaf: colic; externally applied to syphilitic ulcers. Root: constipation	[18, 19, 33, 67, 195, 196]
R12	*R. obtusifolius* L	Broad-leaf dock, bitter dock, blunt-leaf dock	China, Japan, Europe, Africa and Ireland	Whole plant	Nettle, depurative, astringent, constipation, tonic agent, sores, blisters, hyperglycemic, burns, tumors	[62, 193, 194]
R13	*R. patientia* L	Herb patience, garden patience, patience dock, spinach dock	Asia, Europe, North India, Bulgaria and Ukraine	Leaf	Hemostasis, diarrhoea, diarrhoea in cows	[4, 19, 68, 192, 198]
R14	*R. cristatus* DC	Greek dock	France, Turkey and Spain	–	–	[69–71]
R15	*R. vesicarius* L	Bladder dock, country sorrel	South Asia, Egypt and North Africa	Leaf, seed and whole plant	Plant: astringent, antiscorbutic, stomach problems, diuretic. Seed: antidysentery	[18, 72, 73, 203]
R16	*R. luminiastrum* Jaub & Spach		Europe	–	–	[42]
R17	*R. pictus* Forssk	Veined dock	Egypt, Gulf States, Kuwait, Lebanon-Syria, Libya, Palestine, Saudi Arabia, Sinai and Israel	Whole plant	For food	[41, 74, 75] [76, 203]
R18	*R. bucephalophorus* L		North America and Libya	Whole plant	Laxative	[77, 204]
R19	*R. tingitanus* L	Koressa	Europe, Asia and Africa	Whole plant	Hepatoprotective, antidepression, blood purifcation, constipation, tonic	[78, 186]
R20	*R. ecklonianus* Meissner	South African dock	South Africa	Young leaf	Anemia, chlorosis	[79]
R21	*R. abyssinicus* Jacq	Spinach rhubarb, mekmeko	Europe, Africa and Spinach	Young shoot, leaf, fresh or dried plant	Brest cancer, stomach problems, gonorrhea, liver diseases, wounds, diabetes, cough, hypertension, sores, rheumatism, hemorrhoids, scabies, diarrhoea	[80, 123]
R22	*R. confertus* Willd	Russian dock, Asiatic dock, mossy sorrel	Russia, Kazakhstan, China, Hungary, Slovakia, Romania, Italy, Europe, Finland, Norway, Sweden, Lithuania, Britain,Canada, north Dakota,Bulgaria and Ukraine	Leaf, root and rhubarb	Diarrhoea, diarrhoea in cows	[81–91, 198]
R23	*R. hymenosepalus* Torr	Canaigre, canaigre dock, desert rhubarb, wild rhubarb, sand dock	Australia, American California, Sonoran and Mexico	Leaf, tuber and rhubarb	Throat infections	[92, 93, 205, 206]
R24	*R. alpinus* L	Alpine dock, monk's rhubarb	Europe and Asia	Leaf and rhubarb	For food	[94]
R25	*R. rugosus* Campd		North America, Europe	Leaf	For food	[95, 96, 200]
R26	*R. nervosus* Vahl	Ithrib	Himalayas, Nilgiri, Nainital, East Africa and Arab	Leaf	Microbial infections, anticoccidial	[97, 98, 207]
R27	*R. maderensis* Lowe	Azedas, madeira sorrel	Portugal	Leaf	Blood depurative, dermatosis, diuretic, simulated gastrointestinal digestion, antidiabetic	[99, 100]
R28	*R. chinensis* Campd. (Syn. = *R*. *trisetifer*)		Vietnam, China	–	Microbial infections	[101]
R29	*R. algeriensis* Barratte & Murb. (Syn. = *R*. *elongatus*)		Algeria	–	–	[102]
R30	*R. tunetanus*		Tunisia	–	–	[103, 104]
R31	*R*. *rechingerianus* Losinsk. (Syn. = *R*. *pamiricus*)		Trans-Ili Ala-Tau	–	–	[61]
R32	*R*. *lunaria* L		Canarian	–	Diabetes	[16]
R33	*R*. *rothschildianus* Aarons		Palestine	Whole plant	Constipation, diarrhea, wound, diuretic, eczema and for food	[105]
R34	*R*. *sanguineus* L	Bloody dock, red veined dock, red-veined dock, red veined sorrel, red-veined sorrel	America, Canada, Chile and Italy	Young leaf	Wound, bacterial infections and abscesses	[61, 106]
R35	*R*. *acetosella* Linn	Sheep sorrel	Asia and Colombia	Root, the aerial part and leaf	Diuretic, constipation, diaphoretic, antiscorbutic. Fresh plant: urinary and kidney diseases	[18, 195]

– unknown

## Chemical constituents

To date, 268 compounds including 56 quinones (**1**–**56**), 57 flavonoids (**57**–**113**), 25 tannins (**114**–**138**), 6 stilbenes (**139**–**144**), 22 naphthalenes (**145**–**166**), 6 terpenes (**167**–**172**), 3 diterpene alkaloids (**173**–**175**), 14 lignans (**176**–**189**) and 79 other types of components (**190**–**268**) were isolated and reported from 29 *Rumex* species (Table 2).

**Table 2:** The summary of compounds in *Rumex*

No	Compounds	Formula	Species	Plant parts	Ref
Quinones					
**1**	Chrysophanol	C_15_H_10_O_4_	R2, R5, R7, R8, R9, R11, R13, R21, R22, R23, R28, R31	Rh, R, WP, T, A, S, F	[23, 35, 45, 46, 50, 51, 63, 80, 93, 101, 113, 125, 128, 129]
**2**	Chrysophanol-1*-O-β*-D-glucoside	C_21_H_20_O_9_	R8, R31	R, S	[64, 128]
**3**	Chrysophanol-8*-O-β*-D-glucoside (chrysophanein)	C_21_H_20_O_9_	R8, R9, R13, R21, R28	A, S, R, WP	[32, 46, 54, 101, 123, 129, 130]
**4**	Chrysophanol-8*-O-β*-D-galactoside	C_21_H_20_O_9_	R8, R14	R	[52, 112]
**5**	Chrysophanol-1*-O-*(4*-O-β*-D-galactosyl)-*α*-L-rhamnoside	C_27_H_30_O_13_	R2	WP	[184]
**6**	6'-Acetyl-chrysophanol-8-*O*-*β*-D-glucoside	C_23_H_22_O_10_	R8	R	[32, 112, 113]
**7**	Chrysophanol anthrone	C_15_H_12_O_3_	R1	R	[29]
**8**	Emodin (1,6,8-trihydroxy-3-methylanthraquinone)	C_15_H_10_O_5_	R2, R5, R6, R8, R9, R11, R13, R21, R28, R31	Rh, R, WP, A, S, F, L	[23, 32, 34, 35, 40, 45–47, 51, 54, 80, 101, 112, 113, 128, 129]
**9**	Emodin-1*-O-β*-D-glucoside	C_21_H_20_O_10_	R7, R8	R, A	[14, 64]
**10**	Emodin-1*-O-β*-D-glucosyl-*α*-L-rhamnoside	C_27_H_30_O_14_	R5, R31	R, S, L	[128, 131]
**11**	Emodin-6*-O-β*-D-glucoside	C_21_H_20_O_10_	R13	R	[54, 130]
**12**	Emodin-8*-O-β*-D-glucoside (PMEG)	C_21_H_20_O_10_	R4, R6, R8, R9, R13, R28	WP, A, R, S	[23, 32, 34, 38, 46, 47, 101, 112, 129, 130]
**13**	Aloe-emodin	C_15_H_10_O_6_	R2, R8, R13	R, WP, L	[23, 27, 35, 112]
**14**	6-Hydroxy-emodin (citreorosein)	C_15_H_10_O_6_	R9, R21	WP	[50, 123]
**15**	6-Acetoxy-aloe-emodin	C_17_H_12_O_6_	R1	R	[29]
**16**	Emodin dimethylether	C_17_H_14_O_5_	R13	WP	[23]
**17**	Emodin anthrone	C_15_H_12_O_4_	R1	R	[29]
**18**	Physcion (rheochrysin, emodin 3-methyl ether)	C_16_H_12_O_5_	R2, R8, R9, R11, R13, R21, R23, R28	Rh, R, WP, T, A	[23, 35, 46, 50, 51, 54, 80, 93, 101, 113, 129]
**19**	Physcion-8*-O-β*-D-glucoside (physcionin)	C_22_H_22_O_10_	R8, R9, R13, R21, R28	A, F, R, WP	[45, 101, 123, 129, 130]
**20**	Physcion anthrone	C_16_H_14_O_4_	R1	R	[29]
**21**	Rumejaposide A	C_22_H_22_O_11_	R9	R	[26]
**22**	Rumejaposide B	C_22_H_22_O_11_	R9	R	[26]
**23**	Rumejaposide C	C_22_H_22_O_12_	R9	R	[26]
**24**	Rumejaposide D	C_22_H_22_O_13_	R9	R	[26]
**25**	Rumejaposide E	C_21_H_22_O_10_	R7, R9	R	[26, 28]
**26**	Rumejaposide F	C_21_H_22_O_10_	R7, R13	L, R	[27, 28]
**27**	Rumejaposide G	C_21_H_22_O_9_	R7	R	[28]
**28**	Rumejaposide H	C_21_H_22_O_9_	R7	R	[28]
**29**	Rumejaposide I	C_21_H_22_O_10_	R7, R13	L, R	[27, 28]
**30**	Xanthorin-5-methylether	C_17_H_14_O_6_	R13	WP	[23, 24]
**31**	Rumexone	C_17_H_16_O_4_	R6	R	[30]
**32**	Rhein	C_15_H_8_O_6_	R2	R	[35]
** 33**	Rhein-8*-O-β*-D-glucoside	C_21_H_18_O_11_	R9	WP	[50]
** 34**	Cassialoin	C_21_H_22_O_9_	R7, R13	L, R	[27, 28]
** 35**	Phallacinol (telochistin)	C_16_H_12_O_6_	R11	R	[51]
** 36**	1,8-Dihydroxyanthraquinone	C_14_H_8_O_4_	R1	R	[29]
** 37**	Martianine	C_42_H_42_O_17_	R11	R	[132]
** 38**	Rumoside A	C_42_H_42_O_16_	R8, R13	R	[32, 112]
** 39**	10-Hydroxyaloin A	C_21_H_22_O_10_	R8	R	[31]
** 40**	10-Hydroxyaloin B	C_21_H_22_O_10_	R8	R	[31]
** 41**	6-Methoxyl-10-hydroxyaloin A	C_22_H_24_O_11_	R8	R	[32]
** 42**	6-Methoxyl-10-hydroxyaloin B	C_22_H_24_O_11_	R8	R	[32]
** 43**	10-Hydroxycascaroside C	C_27_H_32_O_14_	R11	R	[132]
** 44**	10-Hydroxycascaroside D	C_27_H_32_O_14_	R11	R	[132]
** 45**	Obtusifolate A	C_39_H_42_O_8_	R12	R	[25]
** 46**	Obtusifolate B	C_34_H_34_O_7_	R12	R	[25]
** 47**	Rumexpatientoside A	C_22_H_24_O_10_	R11	R	[133]
** 48**	Rumexpatientoside B	C_22_H_24_O_10_	R11	R	[133]
** 49**	Nepalenside A	C_21_H_22_O_11_	R11	R	[33]
** 50**	Nepalenside B	C_21_H_22_O_11_	R11	R	[33]
** 51**	Helminthosporin	C_15_H_12_O_5_	R21	Rh	[80]
** 52**	1,3,5-Trihydroxy-7-methylanthraquinone	C_15_H_10_O_5_	R13	R	[130]
** 53**	1,5-Dihydroxyanthraquinone	C_14_H_8_O_4_	R6	R	[30]
** 54**	1,3,7-Trihydroxy-6-methylanthraquinone	C_15_H_10_O_5_	R2	WP	[134]
** 55**	Przewalskinone B	C_16_H_12_O_5_	R2	WP	[134]
** 56**	Rumpictusoide A	C_21_H_19_O_10_	R17	WP	[183]
Flavonoids					
** 57**	Vitexin	C_21_H_20_O_10_	R1	A	[57]
** 58**	Isovitexin	C_21_H_20_O_10_	R15	A	[185]
** 59**	Orientin	C_21_H_20_O_11_	R1, R16	A, WP	[42, 57]
** 60**	Acetyl-orientine	C_23_H_22_O_12_	R16	WP	[42]
** 61**	Iso-orientine	C_21_H_20_O_11_	R1	A	[57]
** 62**	Quercetin-3-*O*-*β*-D-galactoside (hyperoside)	C_21_H_20_O_11_	R1, R7, R13, R31	S, R, WP	[36, 44, 47, 49]
** 63**	Kaempferol	C_15_H_10_O_6_	R2, R6, R7, R13	WP, R, A	[14, 23, 34, 35]
** 64**	Kaempferol-3-*O*-*β*-D-glucoside	C_21_H_20_O_11_	R4, R7, R13	WP, A	[14, 23, 36–38]
** 65**	Kaempferol-3-*O*-*α*-L-rhamnoside	C_21_H_20_O_10_	R1, R6	L, WP	[34, 39]
** 66**	Kaempferol-3-*O*-*α*-L-rhamnosyl-(1 → 6)-*β*-D-galactoside	C_27_H_30_O_15_	R5, R7	L, WP	[36, 40]
** 67**	Kaempferol-3*-O-α*-L-arabinosyl-(1 → 6)-*β*-D-galactoside	C_26_H_28_O_15_	R17	A	[41]
** 68**	Kaempferol-3*-O-*[2''-O-acetyl-*α*-L-arabinosyl]-(1 → 6)-*β*-D-galactoside	C_28_H_30_O_16_	R17	A	[41]
** 69**	Kaempferol-7*-O-β*-D-glucoside	C_21_H_20_O_11_	R16	WP	[42]
** 70**	Kaempferol-7*-O-α*-L-rhamnoside	C_21_H_20_O_10_	R16	WP	[42]
** 71**	Quercetin	C_15_H_10_O_7_	R2, R5, R7, R8, R13	F, S, R, A	[14, 35, 45, 47, 48]
** 72**	Quercetin-3*-O-β*-D-glucoside (isoquercetin, ECQ, QGC)	C_21_H_20_O_12_	R4, R5, R7, R13	A, WP, L, S	[14, 23, 27, 37, 38, 46, 47]
** 73**	Quercetin-3*-O-β*-D-glucuronide	C_21_H_18_O_13_	R7, R13	A	[14, 46]
** 74**	Quercetin-3-*β*-D-glucosyl-(1 → 4)-*β*-D-galactoside	C_27_H_30_O_17_	R5	L	[40]
** 75**	Quercetin-3-*O*-*α*-L-rhamnoside (quercitrin)	C_21_H_20_O_11_	R4, R5, R9, R13, R31	L, WP, R, A	[27, 38, 40, 49, 50]
** 76**	Isorhamnetol	C_16_H_12_O_7_	R13	WP,	[23, 37]
** 77**	Isorhamnetol-3-*O*-rutinoside	C_28_H_32_O_16_	R7	WP	[36]
** 78**	Isorhamnetol-3-*O*-*β*-D-galactoside	C_22_H_22_O_12_	R7	WP	[36]
** 79**	Isorhamnetol-3-*O*-*β*-D-glucoside	C_22_H_22_O_12_	R7	WP	[36]
** 80**	Quercetin-3*-O-α*-L-arabinoside	C_20_H_18_O_11_	R4, R16	WP, A	[38, 42, 43]
** 81**	Quercetin-3*-O-α*-L-arabinosyl-(1 → 6)-*β*-D-galactoside	C_26_H_28_O_16_	R17	A	[41]
** 82**	Quercetin-3-*O*-[2''-*O*-acetyl-*α*-L-arabinosyl]-(1 → 6)-*β*-D-galactoside	C_28_H_30_O_17_	R17	A	[41]
** 83**	Quercetin-7*-O-β*-D-glucoside	C_21_H_20_O_12_	R13, R16	S, WP	[42, 44, 47]
** 84**	Quercetin-7*-O-α*-L-rhamnoside	C_21_H_20_O_11_	R16	WP	[42]
** 85**	Rutin	C_21_H_30_O_16_	R5, R8, R31	R, L	[32, 40, 49, 112]
** 86**	5-Hydroxy-4'-methoxyflavone-7*-O-β*-D-rutinoside	C_28_H_32_O_14_	R13	WP	[23, 37]
** 87**	Apigenin	C_15_H_10_O_5_	R1	R	[53]
** 88**	Luteolin (cyanidenon)	C_15_H_10_O_6_	R1, R19, R35	L, WP, A	[136, 186–188]
** 89**	Luteolin-7*-O-β*-D-glucoside	C_21_H_20_O_11_	R16	WP	[42]
** 90**	7-Hydroxy-2,3-dimethyl-chromone	C_11_H_10_O_3_	R14	R	[52]
** 91**	5-Methoxy-7-hydroxy-1(3*H*)-chromone	C_10_H_8_O_4_	R13	R	[53]
** 92**	5,7-Dihydroxy-1(3*H*)-chromone	C_9_H_6_O_4_	R13	R	[53]
** 93**	Mikanin (3,5-dihydroxy-4',6,7-trimethoxyflavone)	C_18_H_16_O_7_	R13	L	[27]
** 94**	3,5-Dihydroxy-6,7,3',4'-tetramethoxyflavone	C_19_H_18_O_8_	R13	L	[27]
** 95**	2,5-Dimethyl-7-hydroxychromone-7*-O-β*-D-glucoside	C_17_H_20_O_8_	R8	R	[31]
** 96**	2,5-Dimethyl-7-hydroxychromone	C_11_H_10_O_3_	R11	R	[51]
** 97**	3-*O*-Methyl quercetin	C_16_H_12_O_7_	R8	F	[45]
** 98**	Tricin-7*-O-β*-D-glucoside	C_23_H_24_O_12_	R22	R	[137]
** 99**	2-(2'-Hydroxypropyl)-5-methyl-7-hydroxychromone	C_13_H_14_O_4_	R13	R	[138]
** 100**	2-(2'-Hydroxypropyl)-5-methyl-7-hydroxychromone-7*-O-β*-D-glucoside	C_19_H_24_O_9_	R13	R	[138]
** 101**	Maackiain	C_16_H_12_O_5_	R13	A	[46]
** 102**	Maackiain-3*-O-β*-D-glucoside	C_22_H_22_O_10_	R13	A	[46]
** 103**	Aloesin	C_19_H_22_O_9_	R11	R	[33]
** 104**	4'-*p*-Acetylcoumaroyl luteolin	C_26_H_18_O_9_	R19	L	[78]
** 105**	Catechin	C_15_H_14_O_6_	R1, R6, R13, R19, R31	R, WP	[34, 49, 53, 54]
** 106**	6-Cl-catechin	C_15_H_13_ClO_5_	R13, R19	R	[54]
** 107**	Epicatechin	C_15_H_14_O_6_	R1, R6, R14, R31	R, WP	[34, 49]
** 108**	(+)-Epigallocatechin	C_15_H_14_O_7_	R1	R	[135]
** 109**	(−)-Epigallocatechin	C_15_H_14_O_7_	R1	R	[135]
** 110**	Epicatechin-3-*O*-gallate	C_22_H_18_O_10_	R1, R31	A, R	[49, 56]
** 111**	Epigallocatechin-3-*O*-gallate	C_22_H_18_O_11_	R1	A	[56]
** 112**	Isokaempferide	C_16_H_12_O_6_	R4	A, R	[148]
** 113**	Quercetin-3,3'-dimethylether	C_17_H_14_O_7_	R4	A, R	[148]
Tannins					
** 114**	Epiafzelechin-(4*β* → 8)-epicatechin-(4*β* → 8)-epicatechin	C_45_H_38_O_17_	R1	A	[56]
** 115**	Epicatechin-(4*β* → 8)-epicatechin-(4*β* → 8)-catechin	C_45_H_38_O_18_	R1	A	[56]
** 116**	Epicatechin-(4*β* → 8)-epicatechin-(4*β* → 8)-epicatechin (Procyanidin C1)	C_45_H_38_O_18_	R1	A	[56]
** 117**	Epicatechin-3-*O*-gallate-(4*β* → 8)-epicatechin-3-*O*-gallate-(4*β* → 8)-epicatechin-3-*O*-gallate	C_66_H_50_O_30_	R1	A	[56]
** 118**	Epicatechin-(4*β* → 8)-epicatechin-(4*β* → 8)-epicatechin-(4*β* → 8)-epicatechin	C_60_H_50_O_24_	R1	A	[56]
** 119**	Epicatechin-3-*O*-gallate-(4*β* → 8)-epicatechin-3-*O*-gallate	C_44_H_34_O_20_	R1	A	[139]
** 120**	Epicatechin-(4*β* → 6)-epicatechin (procyanidin B5)	C_30_H_26_O_12_	R1	A	[56]
** 121**	Epicatechin-(4*β* → 6)-catechin	C_30_H_26_O_12_	R1	A	[56]
** 122**	Epicatechin-(4*β* → 8)-catechin (procyanidin B1)	C_30_H_26_O_12_	R1	A	[56, 107]
** 123**	Catechin-(4*α* → 8)-catechin (procyanidin B3)	C_30_H_26_O_12_	R1	A	[56, 107]
** 124**	Catechin-(4*α* → 8)-epicatechin (procyanidin B4)	C_30_H_26_O_12_	R1	A	[56, 107]
** 125**	Epiafzelechin-(4*β* → 8)-epicatechin (procyanidin B2)	C_30_H_26_O_11_	R1	A	[56, 107]
** 126**	Epicatechin-(4*β* → 8)-epicatechin-3-*O*-gallate	C_37_H_30_O_16_	R1	A	[56]
** 127**	Epiafzelechin-(4*β* → 8)-epicatechin-3-*O*-gallate	C_37_H_30_O_15_	R1	A	[56]
** 128**	Epicatechin-(4*β* → 6)-epicatechin-3-*O*-gallate	C_37_H_30_O_16_	R1	A	[56]
** 129**	Epicatechin-3-*O*-gallate-(4*β* → 6)-epicatechin-3-*O*-gallate	C_44_H_34_O_20_	R1	A	[56]
** 130**	Epiafzelechin-3*-O-*gallate-(4*β* → 8)-epicatechin-3*-O-*gallate	C_44_H_34_O_19_	R1	A	[56]
** 131**	Epicatechin-(2*β* → 7, 4*β* → 8)-epicatechin-(4*β* → 8)-epicatechin (cinnamtannin B1)	C_45_H_36_O_18_	R1	A	[56]
** 132**	Epicatechin-(2*β*- > 7, 4*β* → 8)-epiafzelechin-(4*α* → 8)-epicatechin	C_45_H_36_O_17_	R1	A	[56]
** 133**	Epicatechin-3*-O-*gallate-(2*β* → 7,4*β* → 8)-epicatechin-(4*β* → 8)-epicatechin (cinnamtannin B1-3-*O*-gallate)	C_52_H_40_O_22_	R1	A	[56]
** 134**	Epicatechin-(2*β* → 7, 4*β* → 8)-epicatechin-(4*β* → 8)-phloroglucinol	C_36_H_28_O_14_	R1	A	[56]
** 135**	Epiafzelechin-(4*β* → 6)-epicatechin-3*-O-*gallate	C_37_H_30_O_15_	R1	A	[56]
** 136**	Parameritannin A1	C_60_H_48_O_24_	R1	A	[56]
** 137**	Epicatechin-3-*O*-gallate-(4*β* → 8)-epicatechin-3-*O*-gallate-phloroglucinol	C_50_H_38_O_25_	R1	A	[56]
** 138**	Epicatechin-(2*β* → 7, 4*β* → 8)-epicatechin	C_30_H_26_O_12_	R1	A	[56]
Stilbenoids					
** 139**	Resveratrol	C_14_H_12_O_3_	R2, R8	R, F	[32, 35, 45, 112]
** 140**	(*Z*)-Resveratrol	C_14_H_12_O_3_	R1	R	[124]
** 141**	Polydatin (resveratrol-3-*O*-*β*-D-glucoside, piceid)	C_20_H_22_O_8_	R7, R8	R, A	[14, 32, 112]
** 142**	5,4'-Dihydroxy-3-methoxystilbene	C_15_H_14_O_3_	R18	R	[77]
** 143**	3,5-Dihydroxy-4'-methoxystilbene	C_15_H_14_O_3_	R18	R	[77]
** 144**	5,4'-Dihydroxystilbene-3-*O-α*-arabinoside	C_19_H_20_O_7_	R18	R	[77]
Naphthalenes					
** 145**	Nepodin (musizin)	C_13_H_12_O_3_	R2, R8, R9, R13	R	[32, 35, 112, 113, 130]
** 146**	Nepodin-8*-O-β*-D-glucoside	C_19_H_22_O_8_	R1, R2, R4, R7, R8, R13, R17	R, L, A	[27, 31, 38, 46, 63, 74, 110, 130]
** 147**	Nepodin-8*-O-β*-D-(6'*-O-*acetyl)-glucoside	C_21_H_24_O_9_	R2	R	
** 148**	Neposide	C_19_H_22_O_8_	R2, R22, R24	R, WP	[140, 141]
** 149**	2-Acetyl-3-methyl-6-methoxyl-8-hydroxy-1,4-naphthoquinone	C_14_H_12_O_5_	R9	WP	[141]
** 150**	Torachrysone (TRA, 2-acetyl-1,8-dihydroxy-3-methyl-6-methoxyl-naphthalene)	C_14_H_14_O_4_	R13	WP	[141]
** 151**	Torachrysone-8*-O-β*-D-glucoside	C_20_H_24_O_9_	R2, R7, R9, R13	L, R, A	[27, 46, 53, 63]
** 152**	2-Methoxystypandrone (MSD, 6-acetyl-7-methyl-2-methoxyl-5-hydroxy-1,4-naphthoquinone)	C_14_H_12_O_5_	R9, R10	L, S, R	[115, 116]
** 153**	3-Acetyl-2-methyl-1,5-dihydroxyl-2,3-epoxynaphthoquinol	C_13_H_12_O_5_	R9, R11	R,	[51, 65]
** 154**	Rumexoside	C_20_H_22_O_10_	R2	R	[110]
** 155**	2-Acetyl-4-chloro-1,8-dihydroxy-3-methylnaphthalene-8*-O-β*-D-glucoside (patientoside A)	C_19_H_21_O_8_Cl	R13	R	[117]
** 156**	Patientoside B	C_17_H_18_O_7_Cl_2_	R13	R	[117]
** 157**	4,4''-Binaphthalene-8,8''-*O*,*O*-di-*β*-D-glucoside	C_36_H_42_O_16_	R13	R	[120]
** 158**	6-Hydroxymusizin-8*-O-β*-D-glucopyranoside	C_15_H_14_O_6_	R2	R	[110]
** 159**	3-Acetyl-2-methyl-1,4,5-trihydroxyl-2,3-epoxynaphthoquinol	C_13_H_14_O_5_	R13	R	[118]
** 160**	3-Acetyl-2-methyl-1,5-dihydroxyl-7-methoxyl-2,3-epoxynaphthoquinol	C_14_H_14_O_6_	R13	WP	[119]
** 161**	Rumexone A	C_14_H_18_O_4_	R11	R	[142]
** 162**	Rumexneposide A	C_23_H_26_O_9_	R11	R	[143]
** 163**	Rumexneposide B	C_22_H_26_O_10_	R11	R	[143]
** 164**	Hastatuside B	C_21_H_24_O_9_	R2, R13	L, R	[114] [27]
** 165**	Epi-isoshinanolone	C_11_H_12_O_3_	R13	R	[138]
** 166**	Isoshinanolone	C_11_H_12_O_3_	R9, R13	R, WP	[50, 138]
Terpenes					
** 167**	Tormentic acid	C_30_H_48_O_5_	R9	ST	[121]
** 168**	Myrianthic acid	C_30_H_48_O_6_	R9	ST	[121]
** 169**	2*α*,3*α*,19*α*-Trihydroxy-24-norurs-4(23),12-dien-28-oic acid	C_29_H_44_O_5_	R9	ST	[121]
** 170**	4(*R*),23-Epoxy-2*α*,3*α*,19*α*-trihydroxy-24-norurs-12-en-28-oic acid	C_29_H_44_O_6_	R9	ST	[121]
** 171**	Taraxasterol acetate	C_32_H_52_O_2_	R2	R	[35]
** 172**	Lupeol	C_30_H_50_O	R11	A	[189]
Diterpene alkaloids					
** 173**	7,11,14-Trihydroxy-2,13-dioxohetisane (orientinine)	C_20_H_23_NO_5_	R17	A	[75]
** 174**	6,13,15-Trihydroxyhetisane (acorientine)	C_20_H_27_NO_3_	R17	A	[75]
** 175**	6-Hydroxy-11-deoxy-13-dehydrohetisane (panicudine)	C_20_H_25_NO_3_	R17	A	[75]
Lignans					
** 176**	Arctiin	C_27_H_34_O_11_	R13	WP	[23]
** 177**	3-Hydroxyarctiin	C_27_H_34_O_10_	R13	WP	[23]
** 178**	3-Methoxyarctiin-4''*-O-β*-D-xyloside	C_27_H_34_O_11_	R13	WP	[23]
** 179**	4-Ketopinoresinol	C_20_H_20_O_7_	R13	L	[27]
** 180**	Syringaresinol	C_22_H_26_O_8_	R9, R13	L, WP	[27, 50]
** 181**	Manassantin A	C_42_H_52_O_11_	R13	L	[27]
** 182**	Balanophonin	C_22_H_22_O_7_	R13	L	[27]
** 183**	Schizandriside	C_28_H_32_O_6_	R2	WP	[111]
** 184**	(−)-Isolariciresinol-9*-O-β*-D-xylopyranoside	C_25_H_34_O_10_	R2	WP	[111]
** 185**	(−)-5-Methoxyisolariciresinol-9*-O-β*-D-xylopyranoside	C_26_H_36_O_11_	R2	WP	[111]
** 186**	(+)-5-Methoxyisolariciresinol-9*-O-β*-D-xylopyranoside	C_26_H_36_O_11_	R2	WP	[111]
** 187**	(+)-Lyoniside	C_27_H_38_O_12_	R2	WP	[111]
** 188**	Nudiposide	C_27_H_38_O_12_	R2	WP	[111]
** 189**	(+)-Lyoniresinol-3α*-O-β*-D-glucoside	C_28_H_38_O_13_	R11	R	[33]
Others					
** 190**	Phenylethyl*-O-α*-L-arabinopyransy-(1 → 6)*-O-β*-D-glucoside	C_19_H_28_O_10_	R8	R	[31]
** 191**	Methylorsellinate	C_11_H_14_O_4_	R11	R	[51]
** 192**	Ferulic acid	C_10_H_10_O_4_	R11	R	[51]
** 193**	Methyl 2-acetyl-3,5-dihydroxyphenylacetate	C_11_H_12_O_5_	R11	R	[51]
** 194**	1-(2-Hydroxy-5-methyl-phenyl)-ethanon	C_9_H_10_O_2_	R11	R	[51]
** 195**	Methyl syringate	C_10_H_12_O_5_	R11	R	[51]
** 196**	1-(2,4-Dihydroxy-6-methylphenyl)-ethanon	C_9_H_10_O_3_	R11	R	[51]
** 197**	4-Hydroxybenzene ethanol	C_8_H_10_O_2_	R11	R	[51]
** 198**	Isovanillin	C_8_H_8_O_3_	R11	R	[51]
** 199**	*p*-Coumaricacid-*n*-eicosanyl ester	C_31_H_52_O_3_	R13	S	[47]
** 200**	*Z*-Octadecyl caffeate	C_27_H_44_O_4_	R13	S	[47]
** 201**	Dibutylphthalate	C_16_H_22_O_4_	R11	R	[132]
** 202**	2-Methoxyhydroquinone	C_7_H_8_O_3_	R11	R	[132]
** 203**	Batiansuanmol	C_14_H_18_O_5_	R13	R	[138]
** 204**	Orcinol	C_7_H_8_O_2_	R13	R	[54]
** 205**	*p*-Hydroxybenzoic acid	C_7_H_6_O_3_	R1, R9	L,	[26, 39]
** 206**	*p*-Coumaric acid	C_9_H_8_O_3_	R1, R2, R7	L, R, WP, A	[39, 48, 134, 144]
** 207**	Methyl 3,4-dihydroxyphenylpropionate	C_10_H_12_O_4_	R1	L	[39]
** 208**	Vanillic acid	C_8_H_8_O_4_	R1	L	[39]
** 209**	Isovanillic acid	C_8_H_8_O_4_	R1, R7	L, R	[39, 48]
** 210**	Gallic acid	C_7_H_6_O_5_	R2, R7, R13	R,	[35, 48, 53]
** 211**	Methyl gallate	C_8_H_8_O_5_	R2	R	[35]
** 212**	2,6-Dimethoxy-4-hydroxyl benzoic acid	C_9_H_10_O_5_	R9	A	[26]
** 213**	Pyrocatechin	C_6_H_6_O_2_	R9	A	[145]
** 214**	Syringic acid	C_9_H_10_O_5_	R9	A	[145]
** 215**	3,4-Dihydroxybenzaldehyde	C_7_H_6_O_3_	R9	A	[145]
** 216**	Ethyl 3,4-dihydroxybenzoate	C_9_H_10_O_4_	R9	A	[145]
** 217**	Ethyl gallate	C_9_H_11_O_4_	R9	A	[145]
** 218**	Rumexin	C_15_H_20_O_8_	R4	A	[38]
** 219**	Caffeic acid	C_9_H_8_O_4_	R4	A	[38]
** 220**	1-*O*-caffeoylglucose	C_15_H_18_O_9_	R4	A	[38]
** 221**	1-Methyl caffeic acid	C_10_H_10_O_4_	R4	A	[38]
** 222**	Neochlorogenic acid	C_16_H_18_O_9_	R27	L	[146]
** 223**	(*S*)-4′-Methylnonyl benzoate	C_17_H_26_O_2_	R7	A	[14]
** 224**	5-Methoxy-7-hydroxy-1(3*H*)-benzofuranone	C_9_H_8_O_4_	R11	R	[51]
** 225**	5,7-Dihydroxy-1(3*H*)-benzofuranone	C_9_H_6_O_4_	R13	R	[53]
** 226**	5-Methoxyl-1(3*H*)-benzofuranone-7-glucoside	C_15_H_18_O_9_	R8	R	[31]
** 227**	Sinapic acid	C_11_H_12_O_5_	R1	FL	[147]
** 228**	Protocatechuic acid	C_7_H_6_O_4_	R1	L	[55]
** 229**	*p*-Hydroxycinnamic acid	C_9_H_8_O_3_	R8	R	[190]
** 230**	Streptokordin	C_8_H_9_NO_2_	R11	R	[132]
** 231**	Hastatuside A	C_16_H_18_O_9_	R2	R	[114]
** 232**	*β*-Sitosterol	C_29_H_50_O	R1, R6, R7, R11, R13, R28	A, R, S, L, WP	[34, 39, 47, 48, 53, 101, 189]
** 233**	Daucosterol	C_35_H_60_O_6_	R1, R7, R8, R13, R28	A, R, F, L	[39, 45, 48, 53, 101, 138, 190]
** 234**	Ergosta-6,22-diene-3,5,8-triol	C_28_H_46_O_3_	R21	WP	[123]
** 235**	Nonadecanoic acid-2,3-dihydroxypropyl ester	C_22_H_44_O_4_	R13	R	[53]
** 236**	Hexadecanoic acid 2,3-dihydroxypropyl ester	C_19_H_38_O_4_	R7	R	[48]
** 237**	1-Stearoylglycerol	C_21_H_42_O_4_	R4	A, R	[148]
** 238**	Triacontanol	C_30_H_62_O	R13	S	[47]
** 239**	Dotriacontanol	C_32_H_66_O	R13	S	[47]
** 240**	Hexacosanoic acid	C_26_H_52_O_2_	R6, R13	S, WP	[34, 47]
** 241**	Dotriacontane	C_32_H_66_	R13	S	[47]
** 242**	Glyceryl 1,3-dipalmitate	C_35_H_68_O_5_	R13	S	[47]
** 243**	(2*E*)-8-Hydroxy-2,6-dimethyl-2-octenoic acid	C_10_H_18_O_3_	R11	R,	[132]
** 244**	Tetratriacontane	C_34_H_70_	R13	S	[149]
** 245**	Ceryl alcohol	C_26_H_54_O	R20	A	[125]
** 246**	Oxalic acid	C_2_H_2_O_4_	R1	A	[56]
** 247**	Cardozin	C_10_H_20_O_6_	R7	R	[48]
** 248**	Succinic acid	C_4_H_6_O_4_	R7	R	[48]
** 249**	Sucrose	C_12_H_22_O_11_	R8	R	[190]
** 250**	Rebeccamycin	C_27_H_21_Cl_2_N_3_O_7_	R1	L	[9]
** 251**	Vitamin C	C_6_H_8_O_6_	R1	L	[9]
** 252**	Calcium oxalate	C_2_H_2_O_4_ Ca	R1	L	[9]
** 253**	Tartaric acid	C_4_H_6_O_6_	R1	L	[9]
** 254**	*β*-carotene	C_40_H_56_	R13, R15	R, L	[126, 129]
** 255**	Lutein	C_40_H_56_O_2_	R15, R25	L	[95, 126]
** 256**	Anhydrolutein I	C_40_H_54_O	R25	L	[95]
** 257**	Anhydrolutein II	C_40_H_54_O	R25	L	[95]
** 258**	Riboflavin	C_17_H_20_N_4_O_6_	R13	R	[129]
** 259**	2-*O*-methyl inositol	C_7_H_14_O_6_	R13	A	[46]
** 260**	Stigmasterol	C_29_H_48_O	R13	WP	[23]
** 261**	*α*-Asarone	C_12_H_16_O_3_	R13	WP	[23]
** 262**	7-Hydroxy-5-methoxyphthalide	C_9_H_8_O_4_	R11	R	[51]
** 263**	4-Ethyl heptyl benzoate	C_16_H_24_O_2_	R26	R	[150]
** 264**	Glucosylceramide	C_40_H_77_NO_8_	R12	L	[151]
** 265**	Helonioside A	C_32_H_38_O_17_	R7	R	[48]
** 266**	1*-O-β*-D-(2,4-dihydroxy-6-methoxyphenyl)-6*-O-*(4-hydroxy-3,5-dimethoxybenzoyl)-glucoside	C_22_H_26_O_13_	R1	A	[56]
** 267**	1-*O*-*β*-D-(3,5-Dimethoxy-4-hydroxyphenol)-(6-*O*-galloyl)-glucoside	C_21_H_24_O_13_	R11	R	[33]
** 268**	RA-P (Polysaccharide (D-glucose-—-D-arabinose)		R1	R	[127]

*Rh* rhizomes, *R* roots, *WP* whole plants, *T* tubers, *A* the aerial part, *S* seeds, *L* leaves, *F* fruits, *S *stems, *FL* flowers

### Quinones

**Fig. 1:**
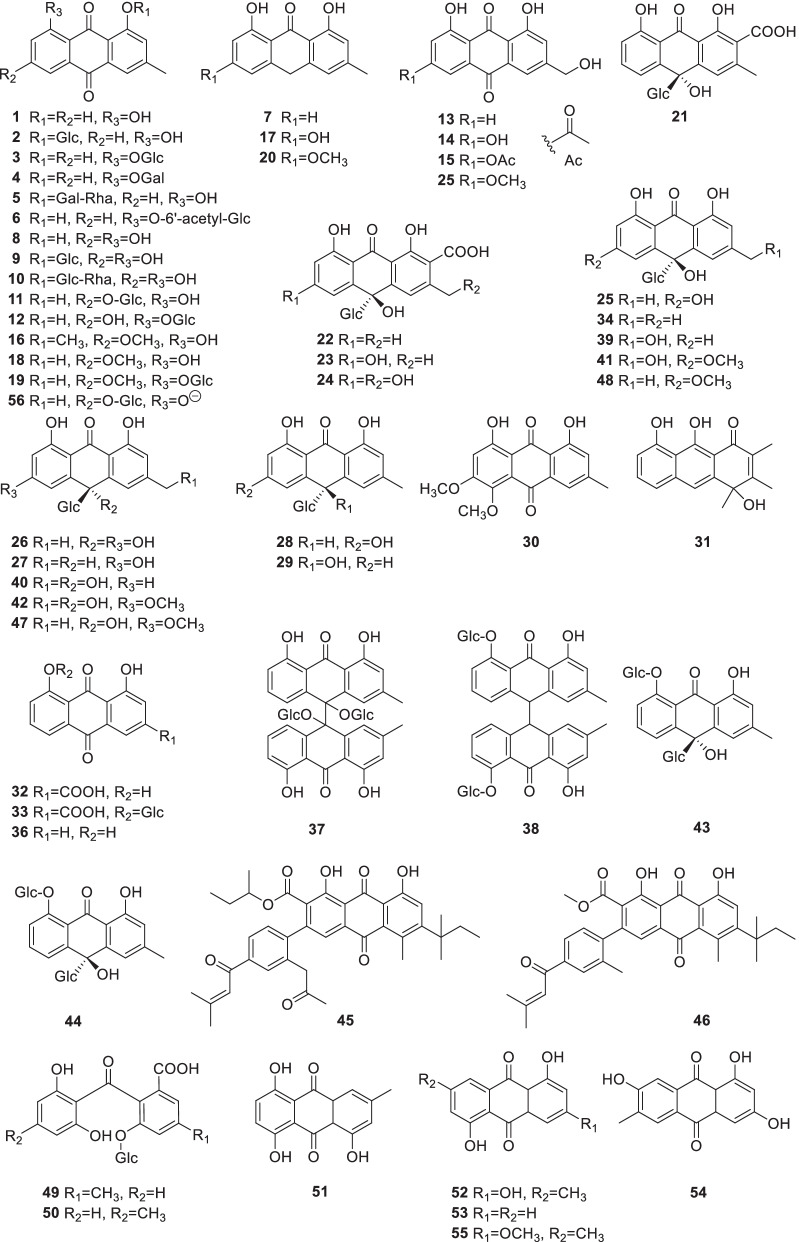
Structures of quinones (**1**–**56**)

Quinones are widely found in *Rumex*, particularly accumulated in the roots. 56 quinones (Fig. 1) including anthraquinones, anthranones, and *seco*-anthraquinones and their glycosides and dimers were isolated and identified from more than 17 *Rumex* species (Table 2). Among them, anthraquinone *O*- and *C*- glycosides with glucose, galactose, rhamnose, and 6-hydroxyacetylated glucose as commonly existing sugar moieties, were normally found in *Rumex*. Three anthraquinones, chrysophanol **(1**), emodin (**8**) and physcion (**18**) are commonly used indicators to evaluate the quality of *Rumex* plants [22]. Some new molecules were also reported. For example, xanthorin-5-methylether (**30**) was isolated from *R*. *patientia* for the first time [23, 24], and two new antioxidant anthraquinones, obtusifolate A (**45**) and B (**46**) were isolated from *R*. *obtusifolius* [25].

The anthranones often existed in pairs of enantiomers, whose *meso*-position is commonly connected with a *C*-glycosyl moiety. The enantiomers, rumejaposides A (**21**) and B (**22**), E (**25**) and F (**26**), G (**27**) and H (**28**) were reported from *R*. *dentatus*, *R*. *japonicus*, *R*. *nepalensis* and *R*. *patientia* [26–28]. Three hydroxyanthrones, chrysophanol anthrone (**7**), emodin anthrone (**17**), physcion anthrone (**20**), whose C-10 were reduced as an alphatic methylene, were isolated from the roots of *R*. *acetosa* for the first time [29], while a new anthrone, rumexone (**31**) was reported from the roots of *R*. *crispus* [30]. Two anthranones, 10-hydroxyaloins A (**39**) and B (**40**) were reported from *Rumex* for the first time [31]. A new 8-ionized hydroxylated 9,10-anthraquinone namely, rumpictusoide A (**56**) was isolated from the whole plant of *R*. *pictus* [183]. Moreover, two new oxanthrone *C*-glucosides 6-methoxyl-10-hydroxyaloins A (**41**) and B (**42**) were isolated from the roots of *R*. *gmelini* [32].

*Seco*-anthraquinones are oxidized anthraquinones with a loop opened at C-10, resulting in the fixed planar structure of anthraquinone destroyed and causing of a steric hindrance between the two left benzene rings. So far, only two *seco*-anthraquinone glucosides, nepalensides A (**49**) and B (**50**) were reported from the roots of *R*. *nepalensis* [33].

### Flavonoids

**Fig. 2:**
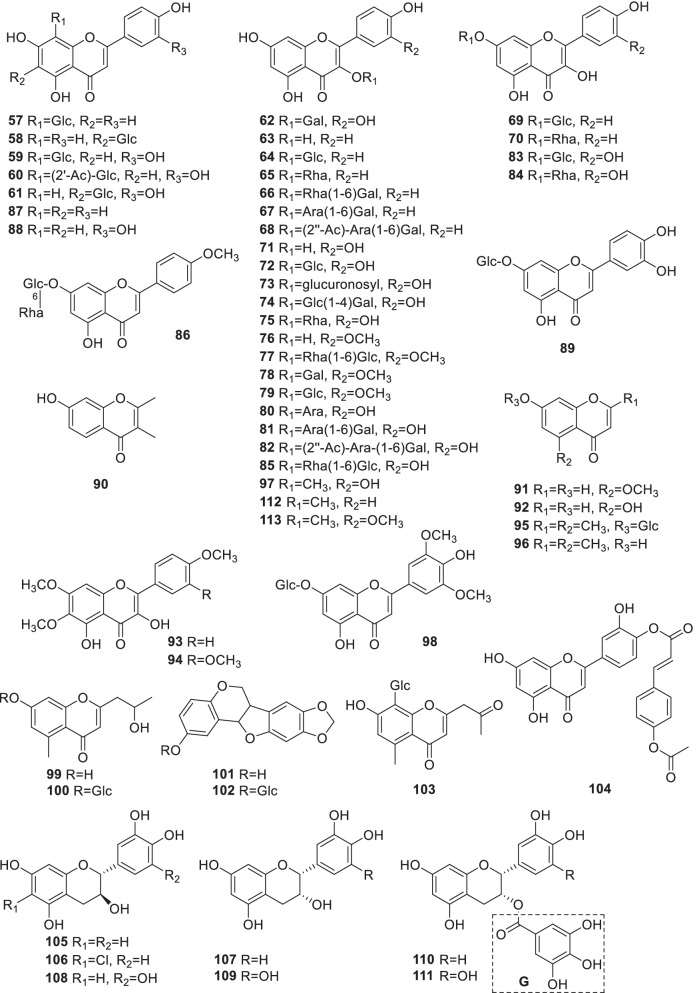
Structures of flavonoids (**57–113**)

Flavonoids are one of the most important bioactive components existing widely in plant kingdom. To date, 57 flavonoids (**57**–**113**) including flavones, flavanols, chromones and their

glycosides were reported from *Rumex* (Fig. 2, Table 2). They are mostly derived from kaempferol (**63**) and quercetin (**71**) connecting with glucosyl, rhamnosyl, galactosyl and arabinosyl moieties at different positions. For example, kaempferol (**63**) together with seven glycosides, -3-*O*-*β*-D-glucoside (**64**), -3-*O*-*α*-L-rhamnoside (**65**), -3-*O*-*α*-L-rhamnosyl-(1 → 6)-*β*-D-galactoside(**66**), -3-*O*-*α*-L-arabinosyl-(1 → 6)-*β*-D-galactoside (**67**), -3-*O*-(2''-*O*-acetyl-*α*-L-arabinosyl)-(1 → 6)-*β*-D-galactoside (**68**), -7-*O*-*β*-D-glucoside (**69**) and -7-*O*-*α*-L-rhamnoside (**70**) [14, 23, 34–42], and quercetin (**71**) together with 11 derivatives, -3-*O*-*β*-D-glucoside (**72**), -3-*O*-*β*-D-glucuronide (**73**), -3-*O*-*β*-D-glucosyl(1 → 4)-*β*-D-galactoside (**74**), -3-*O*-*α*-L-rhamnoside (**75**), -3-*O*-*α*-L-arabinoside (**80**), -3-*O*-*α*-L-arabinosyl-(1 → 6)-*β*-D-galactoside (**81**), -3-*O*-[2''-*O*-acetyl-*α*-L-arabinosyl]-(1 → 6)-*β*-D-galactoside (**82**), -7-*O*-*β*-D-glucoside (**83**), -7-*O*-*α*-L-rhamnoside (**84**), 3-*O*-methyl quercetin (**97**) and -3,3'-dimethylether (**113**) [14, 23, 27, 35, 37, 38, 40–50, 148], were reported from several *Rumex* plants.

Moreover, a new chromone glucoside, 2,5-dimethyl-7-hydroxychromone-7-*O*-*β*-D-glucoside (**95**) was isolated from the root of *R*. *gmelini* [31], and five chromones, 7-hydroxy-2,3-dimethyl-chromone (**90**), 5-methoxy-7-hydroxy-1(3*H*)-chromone (**91**), 5,7-dihydroxy-1(3*H*)-chromone (**92**), 2,5-dimethyl-7-hydroxychromone-7-*O*-*β*-D-glucoside (**95**) and 2,5-dimethyl-7-hydroxychromone (**96**) were reported from *R*. *gmelini*, *R*. *nepalensis*, *R*. *patientia* and *R*. *cristatus* [31, 51–53].

Catechin (**105**) and epicatechin (**107**) are commonly distributed in *R*. *patientia*, the roots of *R*. *rechingerianus*, the whole plant of *R*. *crispus*, and the leaves of *R*. *acetosa* [34, 37, 39, 49, 54, 55]. Moreover, a variety of flavan-3-ols, **105**, **107**, epicatechin-3-*O*-gallate (**110**), epigallocatechin-3-*O*-gallate (**111**) were isolated from *R*. *acetosa* [49, 56].

### Tannins

**Fig. 3:**
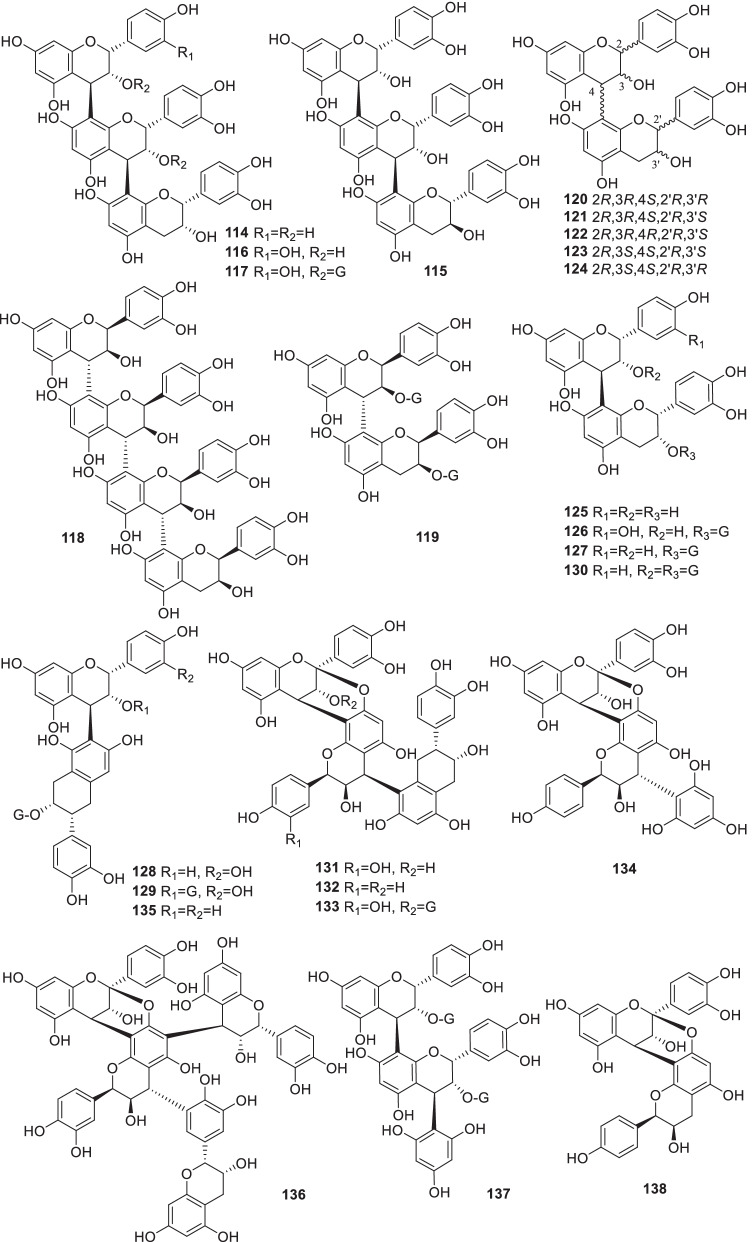
Structures of tannins (**114–138**)

Tannins, which may be involved with the hemostasis activity, are abundant in *Rumex* plants. So far, 25 condensed tannins (**114**–**138**) (Fig. 3, Table 2) were reported from the genus *Rumex*.

Chemical investigations on the EtOAc fraction of acetone–water extract of the aerial parts of *R*. *acetosa* showed that *R*. *acetosa* was rich in tannins. Five new condensed tannin dimers, epiafzelechin-(4*β* → 8)-epicatechin-3-*O*-gallate (**127**), cinnamtannin B1-3-*O*-gallate (**132**) and epiafzelechin-(4*β* → 6)-epicatechin-3-*O*-gallate (**135**), and trimers, epiafzelechin-(4*β* → 8)-epicatechin-(4*β* → 8)-epicatechin (**114**), and epicatechin-(2*β* → 7, 4*β* → 8)-epiafzelechin-(4*α* → 8)-epicatechin (**132**), were reported. In addition, some procyanidins and propelargonidins, epiafzelechin-(4*β* → 8)-epicatechin-(4*β* → 8)-epicatechin (**114**), epicatechin-(4*β* → 8)-epi-catechin-(4*β* → 8)-catechin (**115**), procyanidin C1 (**116**), epicatechin-(4*β* → 6)-catechin (**121**), procyanidin B1-B5 (**120**, **122–125**), and epicatechin-(4*β* → 8)-epicatechin-3-*O*-gallate (**126**), were also isolated [56, 107].

### Stilbenes

**Fig. 4:**
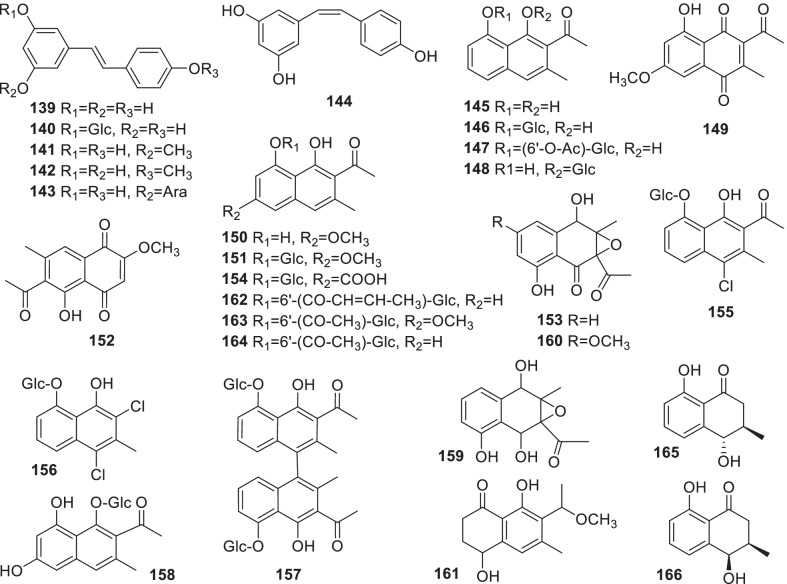
Structures of stilbenes (**139–144**) and naphthalenes (**145–166**)

So far, 6 stilbenes have been separated from *Rumex* (**139**–**144**) (Fig. 4, Table 2). Resveratrol (**139**) isolated from *R*. *japonica* Houtt was found for the first time from the Polygonaceae family [108]. It has been widely applied in cardiovascular protection and as antioxidation agent [109]. Resveratrol (**139**), (*Z*)-resveratrol (**140**) and polydatin (**141**) were obtained from *Rumex* spp. [14, 32, 35, 45, 110, 111]. 5,4'-Dihydroxy-3-methoxystilbene (**142**), 3,5-dihydroxy-4'-methoxystilbene (**143**) and 5,4'-dihydroxy-stilbene-3-*O-α*-arabinoside (**144**) were separated from the roots of *R*. *bucephalophorus* [77].

### Naphthalenes

Naphthalenes are also widely distributed in *Rumex*. At present, 22 naphthalenes including naphthol, *α*-naphthoquinones and their derivatives have been identified from *Rumex* (**145**–**166**) (Fig. 4, Table 2). Nepodin (**145**) and nepodin-8-*O*-*β*-D-glucoside (**146**) are widespread in *Rumex* [31, 45, 112, 113]. In addition, **145**, nepodin-8-*O*-*β*-D-(6'-*O*-acetyl)-glucoside (**147**), rumexoside (**154**), 6-hydroxymusizin-8-*O*-*β*-D-glucopyranoside (**158**) and hastatuside B (**164**) were isolated from *R*. *hastatus* [35, 110, 114]. 2-Methoxystypandrone (**152**) was isolated from *R*. *japonicus* and *R*. *maritimus* [115, 116]. Notably, some naphthalenes containing Cl, 2-acetyl-4-chloro-1,8-dihydroxy-3-methylnaphthalene-8-*O*-*β*-D-glucoside (**155**) and patientoside B (**156**) were isolated from *R*. *patientia* [117]. Moreover, 3-acetyl-2-methyl-1,5-dihydroxyl-2,3- epoxynaphthoquinol (**153**), 3-acetyl-2-methyl-1,4,5-trihydroxyl-2,3-epoxy-naphtho-quinol (**159**) and 3-acetyl-2-methyl-1,5-dihydroxyl-7-methoxyl-2,3-epoxynaphthoquinol (**160**), which contain the ethylene oxide part of the structure, were rarely found in *Rumex*, and they were reported from *R*. *patientia*, *R*. *japonicus* and *R*. *nepalensis* [51, 65, 118, 119]. 4,4''-Binaphthalene-8,8''-*O*,*O*-di-*β*-D-glucoside (**157**) was isolated from *R*. *patientia* [120].

### Terpenes

**Fig. 5:**
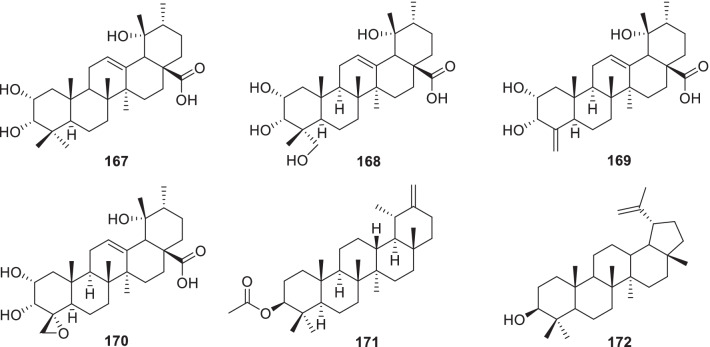
Structures of terpenes (**167–172**)

Until now, only six terpenes have been reported from *Rumex* (Fig. 5, Table 2). Four pentacyclic triterpenes, i.e., tormentic acid (**167**), myrianthic acid (**168**) and 2*α*,3*α*,19*α*-trihydroxy-24-norurs-4(23), 12-dien-28-oic acid (**169**) and (4*R*)-23-epoxy-2*α*,3*α*,19*α*-trihydroxy-24-norurs-12-en-28-oic acid (**170**) were obtained from the EtOAc fraction of the stems of *R*. *japonicus*. Of them, **169** and **170** were two new 24-norursane type triterpenoids, whose C-12 and C-13 were existed as double bonds [121]. A ursane (*α*-amyrane) type triterpene, taraxasterol acetate (**171**) was isolated from *R*. *hastatus*. [63]. And lupeol (**172**) was isolated from the roots of *R*. *nepalensis* for the first time [122].

### Diterpene alkaloids

**Fig. 6:**
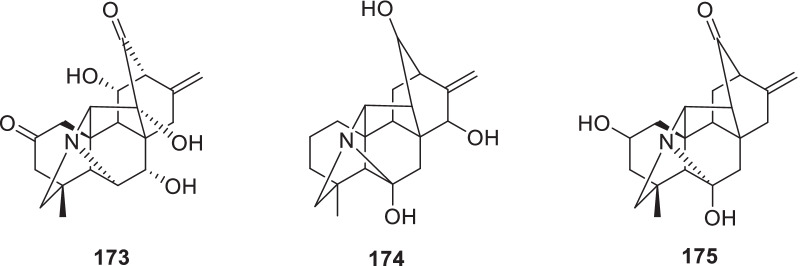
Structures of diterpene alkaloids (**173–175**)

So far, only three hetisane-type (C-20) diterpene alkaloids, orientinine (7,11,14-trihydroxy-2,13-dioxohetisane, **173**), acorientine (6,13,15-trihydroxyhetisane, **174**) and panicudine (6-hydroxy-11-deoxy-13-dehydrohetisane, **175**) were reported from the aerial part of *R*. *pictus*. They might be biosynthesized from tetra- or penta-cyclic diterpenes [75] (Fig. 6, Table 2).

### Lignans

**Fig. 7:**
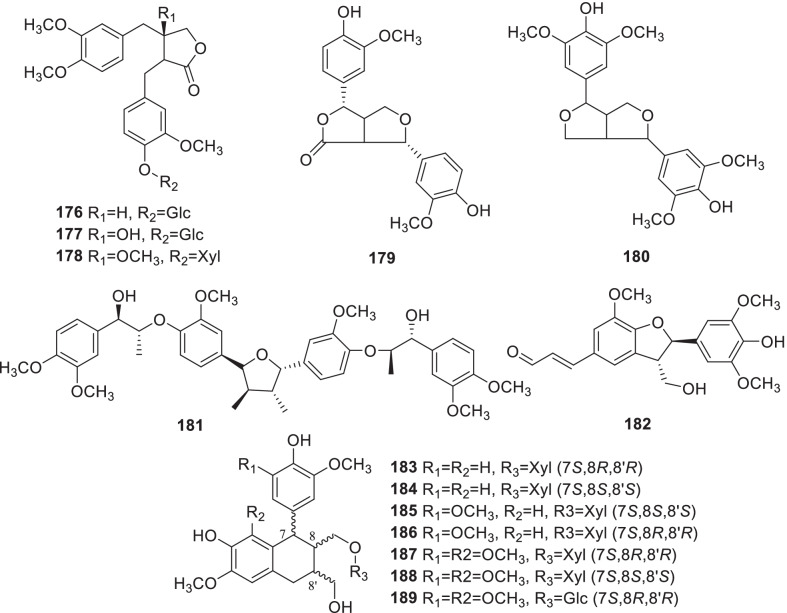
Structures of lignans (**176–189**)

Fourteen lignans (**176**–**189**) were summarized from *Rumex* (Fig. 7, Table 2). A new lignan, 3-methoxyarctiin-4''-*O*-*β*-D-xyloside (**178**), and two known ones, arctiin (**176**) and 3-hydroxy-arctiin (**177**), were obtained from *R*. *patientia* [23]. Six lignan glycosides, schizandriside (**183**), (-)-isolariciresinol-9-*O*-*β*-D-xylopyranoside (**184**), (-)-5-methoxyisolariciresinol-9-*O*-*β*-D-xylopyranoside (**185**), (+)-5-methoxyisolariciresinol-9-*O*-*β*-D-xylopyranoside (**186**), (+)-lyoniside (**187**) and nudiposide (**188**) were reported from *R*. *hastatus* for the first time [111].

### Other compounds

**Fig. 8:**
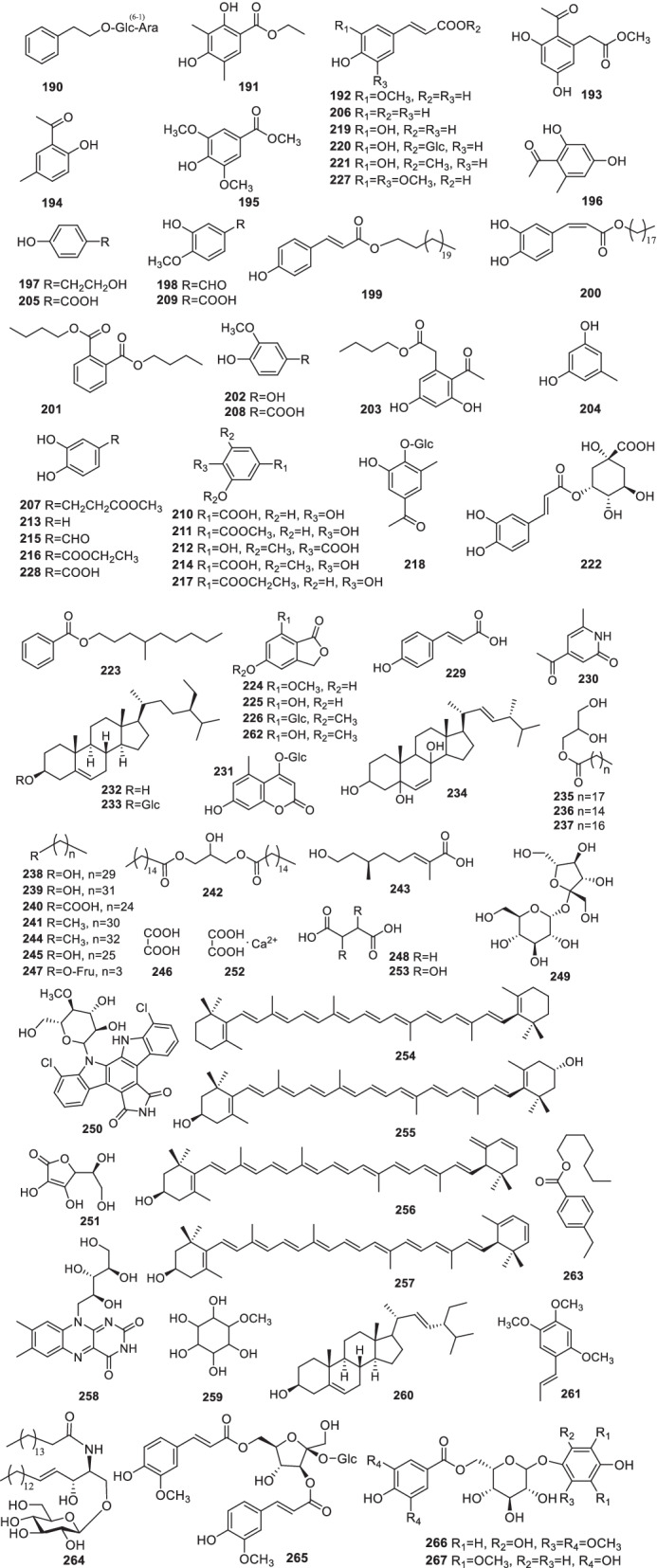
Structures of other compounds (**190–268**) (Note**:268** not given)

Up to now, 79 coumarins, sterides, alkaloids, glycosides and polysaccharide were found in *Rumex* (**190**–**268**) (Fig. 8, Table 2). Phenylethyl-*O*-*α*-L-arabinopyransy-(1 → 6)-*O*-*β*-D-glucoside (**190**) and 5-methoxyl-1(3*H*)-benzofuranone-7-glucoside (**226**) were isolated from *R*. *gmelini* for the first time [31]. *p*-Hydroxybenzoic acid (**205**), *p*-coumaric acid (**206**), methyl 3,4-dihydrophenylpropionate (**207**), vanillic acid (**208**) and isovanillic acid (**209**) were isolated from the leaves of *R*. *acetosa* [39]. *β*-Sitosterol (**232**) and daucosterol (**233**) are commonly distributed in *R*. *acetosa*, *R*. *chinensis*, *R*. *crispus* and *R*. *gmelini* [31, 34, 39, 101]. 2,6-Dimethoxy-4-hydroxyl benzoic acid (**212**) was isolated from *R*. *japonicus* [26]. Moreover, rumexin (**218**), caffeic acid (**219**), 1-*O*-caffeoylglucose (**220**) and 1-methyl caffeic acid (**221**) were isolated from the aerial parts of *R*. *aquatica* [38]. Recently, one new compound (*S*)-4′-methylnonyl benzoate (**223**) was reported from *R*. *dentatus* [14]. Ergosta-6,22-diene-3,5,8-triol (**234**) was isolated from the EtOAc fraction of *R*. *abyssinicus* for the first time [123]. Conventional techniques and supercritical fluid extraction (SFE) were compared and the latter yielded great efficiency of phenolics from the roots of *R*. *acetosa* [124].

Ceryl alcohol (**245**) from *R*. *ecklonianus* [125], and *β*-carotene (**254**) and lutein (**255**) from *R*. *vesicarius* [126] were reported. Moreover, anhydroluteins I (**256**) and II (**257**) were separated from *R*. *rugosus* together with **255** [95]. From the roots of *R*. *dentatus*, helonioside A (**265**) was isolated for the first time [48]. One new phloroglucinol glycoside 1-*O*-*β*-D-(2,4-dihydroxy-6-methoxyphenyl)-6-*O*-(4-hydroxy-3,5-dimethoxybenzoyl)-glucoside (**266**) was isolated from *R*. *acetosa* [56]. It was the first time that 1-*O*-β-D-(3,5-dimethoxy-4-hydroxyphenol)-(6-*O*-galloyl)-glucoside (**267**) was isolated from *R*. *nepalensis* [33].

*Rumex* polysaccharides have rarely been studied, and only one polysaccharide, RA-P (**268**), which has a 30 kDa molecular weight and consists of D-glucose and D-arabinose, was reported from *R*. *acetosa* [127].

## LC–MS analysis

The chemical compositions of *Rumex* spp. were also analyzed by LC–MS techniques. Untargeted metabolomic profiling via UHPLC-Q-TOF–MS analysis on the flowers and stems of *R*. *tunetanus* resulted in the identification of 60 compounds, 18 of which were reported from the Polygonaceae family for the first time. Quercetin-3-*O*-*β*-D-glucuronide (**73**) was found to be the most abundant phenolic compound in flowers and epicatechin-3-*O*-gallate (**110**) in stems [103]. Moreover, 44 bioactive components classified as sugars, flavanols, tannins and phenolics were clarified from the flowers and stems of *R*. *algeriensis* based on RP-HPLC–DAD-QTOF-MS and MS–MS [102]. The analysis of sex-related differences in phenolics of *R*. *thyrsiflorus* has shown female plants of *R*. *thyrsiflorus* contain more bioactive components than males, such as phenolic acids and flavonoids, especially catechin (**105**) [20].

## Bioactivity

*Rumex* has been used as food and medicine in the folk. In addition to important role of *Rumex* in the traditional application, during the past few decades, it was subjected to scientific investigations of the structure of isolated chemical components and their clinical applications by several research groups. Pharmacological studies on *Rumex* extracts and its pure components revealed a wide range of bioactivities, involving antimicrobial, anti-inflammatory, antiviral, renal and gastrointestinal protective effects, antioxidant, antitumor and anti-diabetes effects.

### Antimicrobial

Bioassay-guided isolation on the whole plants of *R*. *abyssinicus* yielded six antimicrobial quinones, chrysophanol (**1**) and its 8*-O-β*-D-glucoside (**3**), emodin (**8**), 6-hydroxyemodin (**14**), physcion (**18**) and its 8-*O*-*β*-D-glucoside (**19**), with MIC values of 8—256 μg/mL [123].

Proanthocyanidin-enriched extract from the aqueous fraction of the acetone–water (7: 3) extract of the aerial parts of *R*. *acetosa* (5 μg/mL—15 μg/mL) could interfered with the adhesion of *Porphyromonas gingivalis* (ATCC 33,277) to KB cells (ATCC CCL-17) both in vitro and in situ. In silico docking assay, a main active constituent from *R*. *acetosa*, epiafzelechin-3-*O*-gallate-(4*β* → 8)-epicatechin-3-*O*-gallate (**130**) exhibited the ability to interact with the active side of Arg-gingipain and the hemagglutinin from *P*. *gingivalis* [139].

A bacteriostasis experiment of two naphthalenes, torachrysone (**150**) and 2-methoxy-stypandrone (**152**) isolated from *R. japonicus* roots, showed inhibitory effect on both gram-negative and gram-positive bacteria [152]. The antibacterial (*Bacillus subtilis*, *Escherichia coli*, *Moraxella catarrhalis*, etc.) potential of the *n*-hexane, chloroform, aqueous fractions of 14 *Rumex* from Carpathian Basin (*R*. *acetosella*, *R*. *acetosa*, *R*. *alpinus*, *R*. *aquaticus*, *R*. *crispus*, *R*. *patientia*, *R*. *pulcher*, *R. conglomeratus*, *R*. *thyrsiflorus*, etc.) were investigated by the disc diffusion method. It showed that the *n*-hexane and chloroform fractions of roots of *R. acetosa*, *R. alpinus*, *R. aquaticus*, *R. conglomeratus* and *R. patientia* exhibited stronger activity against bacteria (inhibition zones > 15 mm). Naphthalenes (**145**, **146**, **151**, **152**) exhibited antibacterial capacity against several bacterial strains (MIC = 48—57.8 μM, in case of *M*. *catarrhalis*; MIC = 96—529.1 μM, in case of *B*. *subtilis*) than anthraquinones (**1**, **3**, **8**, **12**, **14**, **18**), flavonoids **(62**, **71**, **80**, **105, 112, 113**), stilbenes (**139**, **141**) and 1-stearoylglycerol (**237**), etc., which were isolated from *R. aquaticus* [148].

Antimicrobial study demonstrated that *R*. *crispus* and *R*. *sanguineus* have the potential for wound healing due to their anti-*Acinetobacter baumannii* activities (MIC = 1.0—2.0 mg/mL, *R*. *crispus*; 1.0—2.8 mg/mL, aerial parts of *R*. *sanguineus*; 1.4—4.0 mg/mL, roots of *R*. *sanguineus*) [106].

### Anti-inflammatory

The potential effects of anti-inflammatory of AST2017-01 composing of processed *R. crispus* and *Cordyceps militaris* which was widely used in folk medicines in Korea, as well as chrysophanol (**1**) on the treatment of ovalbumin-induced allergic rhinitis (AR) rats were investigated. The serum and tissue nasal mucosa levels of IgE, histamine, TSLP, TNF-*α*, IL-1, IL-4, IL-5 and IL-13 were both decreased by treatment with AST2017-01 and **1** (positive control: dexamethasone), indicating that *R. crispus* and **1** has the ability to prevent and treat AR [153]. The aqueous extract of roots of *R. patientia* has anti-inflammatory action in vivo. The higher dose of extract (150 mg/kg) showed inhibition (41.7%) of edema in rats compared with the positive control, indomethacin (10 mg/kg, 36.6%) [21]. Methanolic extracts of the roots and stems of *R*. *roseus* exhibited anti-inflammatory functions in intestinal epithelial cells, reducing TNF-*α*-induced gene expression of IL-6 and IL-8 [154].

The ethanol extract of the roots of *R*. *japonicus* could be a therapeutic agent for atopic dermatitis. Skin inflammation in Balb/c mice was alleviated with the extract in vivo. Moreover, an in vitro experiment showed that the extract of *R*. *japonicus* decreased the phosphorylation of MAPK and stimulated NF-*κ*B in TNF-*α* in HaCaT cells [155]. The methanolic extract of *R*. *japonicus* inhibited dextran sulfate sodium (DSS)-induced colitis in C57BL/6 N mice by protecting tight junction connections in the colonic tissue. It was observed that *R*. *japonicus* has the potential to treat colitis [156]. Ethyl acetate extract of the roots of *R*. *crispus* showed anti-inflammatory activity in inhibiting NO production and decreasing the secretion of proinflammatory cytokines [157].

### Antivirus

1,4-Naphthoquinone and naphthalenes from *R. aquaticus* presented antiviral activity against *herpes simplex* virus type 2 (HSV-2) replication infected Vero cells. In which, musizin (**145**) showed dose dependent inhibitory property, causing a 2.00 log_10_ reduction in HSV-2 at 6.25 μM, on a traditional virus yield reduction test and *q*PCR assay. It suggested that *R. aquaticus* had the potential to treat HSV-2 infected patients [158].

Acetone–water extract (R2, which contains oligomeric, polymeric proanthocyanidins and flavonoids) from the aerial parts of *R*. *acetosa* showed obvious antiviral activities via plaque reduction test and MTT assay on Vero cells. R2 was 100% against herpes simplex virus type-1 at concentrations > 1 μg/mL (IC_50_ = 0.8 ± 0.04 μg/mL). At concentrations > 25 μg/mL (CC_50_ = 78.6 ± 12.7 μg/mL), cell vitality was more than 100% reduced by R2 [107].

### The function in kidney and gastrointestinal tract

It is noted that quercetin-3*-O-β*-D-glucoside (**72**, QGC) from *R. aquaticus* could alleviate the modle that indomethacin (nonsteroidal anti-inflammatory drugs) induced gastric damage of rats and ethanol extract of *R. aquaticus* had a protective effect on the inflammation of gastric epithelial cells caused by *Helicobacter pylori*. In vivo research suggested that QGC pretreatment could decrease gastric damage by increasing mucus secretion, downregulating the expression of intercellular adhesion molecule-1 and decreasing the activity of myeloperoxidase. The in vitro test found that flavonoids including QGC could inhibit proinflammatory cytokine expression and inhibit the proliferation of an adenocarcinoma gastric cell line (AGS) [159, 160]. The cytoprotective effect of QGC against hydrogen peroxide-induced oxidative stress was noticed in AGS [161]. Moreover, QGC also showed protective efficiency in a rat reflux esophagitis model in a dose-dependent manner (1—30 mg/kg) [162].

Ten anthraquinones chrysophanol (**1**), chrysophanol-8-*O*-*β*-D-glucoside (**3**), 6'-acetyl-chrysophanol-8-*O*-*β*-D-glucoside (**6**), emodin (**8**), emodin-8-*O*-*β*-D-glucoside (**12**), physcion (**18**), aloe-emodin (**13**), rumexpatientosides A (**47**) and B (**48**) and nepalenside A (**49**) from *R*. *patientia*, *R*. *nepalensis*, *R*. *hastatus* not only inhibited the secretion of IL-6, but also decreased collagen IV and fibronectin production at a concentration of 10 µM in vitro. On which concentration, they were nontoxic to cells [133]. It suggested that anthraquinones have great potential to treat kidney disease.

### Antioxidant properties

An extraction technology to obtain the total phenolics of *R. acetosa* was optimized and the antioxidant activity of different plant parts of *R. acetosa* was well investigated. It was found that the 80% methanol extract of the roots (IC_50_ = 118.8 μM) showed higher scavenging activity to DPPH free radicals than the other parts (leaves: IC_50_ = 201.6 μM, flowers and fruits: IC_50_ = 230.1 μM, stems: IC_50_ = 411.2 μM) [163]. The roots of *R. thyrsiflorus* [164], ethanol extracts of *R. obtusifolius* and *R. crispus* showed antioxidant ability on DPPH, ABTS^+^ and FRAP assays [165]. Moreover, *R*. *tingitanus* leaves, *R*. *dentatus*, *R*. *rothschildianus* leaves, *R*. *roseus* and *R*. *vesicarius* also showed antioxidant activity on DPPH assay [13, 78, 105, 154, 166, 167]. Phenolics isolated from *R*. *tunetanus* flowers and stems displayed antioxidant properties on DPPH and FRAP assays [103]. DPPH, ABTS^+^, NO_2_^−^ radical scavenging and phosphomolybdate antioxidant assays verified that *R*. *acetosella* has antioxidant properties [168]. Phenolic constitutions from *R. maderensis* dispalyed antioxidant activity after the gastrointestinal digestion process. These components are known as dietary polyphenols and have the potential to be developed as functional products [99].

Moreover, the total antioxidant capacities of *R. crispus* were found to be 49.4%—86.4% on DPPH, ABTS^+^, NO, phosphomolybdate and SPF assays, which provided the basis to develop *R. crispus* as antioxidant, antiaging and skin care products [169]. Later on, the ripe fruits of *R. crispus* were studied and the aqueous extract showed antioxidant activity in vitro [170]. Dichloromethane and ethyl acetate extracts of *R. crispus* exhibited stronger antioxidant activity, which were associated with the concentration of polyphenols and flavonoids [157]. The antioxidant activities of chrysophanol (**1**), 1,3,7-trihydroxy-6-methylanthraquinone (**54**), przewalskinone B (**55**) and *p*-coumaric acid (**206**) isolated from *R*. *hastatus* were investigated on a nitric oxide radical scavenging assay, whose IC_50_ values were 0.39, 0.47, 0.45, and 0.45 mM, respectively [134].

### Antitumor properties

MTT assays on HeLa (human cervical carcinoma), A431 (skin epidermoid carcinoma) and MCF7 (human breast adenocarcinoma) cell lines showed that *R. acetosa* and *R. thyrsiflorus* could inhibit the tumor cell proliferation [171]. The fruit of *R*. *crispus* showed cytotoxicity on HeLa, MCF7 and HT-29 (colon adenocarcinoma) cells in vitro [170]. The methanolic extract of *R*. *vesicarius* was assessed for hepatoprotective effects in vitro. CCl_4_-induced hepatotoxicity was observed at 100 mg/kg bw and 200 mg/kg bw. The plant also has cytotoxicity in HepG2 (human hepatoma cancer) cell lines [172]. Dichloromethane extract of *R*. *crispus* roots inhibited the growth and induced cellular apoptosis of HepG2 cells [157]. The hexane fraction of *R*. *rothschildianus* leaves showed 98.9% and 97.4% inhibition of HeLa cells and MCF7 cells at a concentration of 4 mg/mL [105].

Different plant parts (stems, roots, flowers and leaves) of *R. vesicarius* were screened for their cytotoxicity by the MTS method on MCF7, Lovo and Caco-2 (human colon cancer), and HepG2 cell lines. The stems displayed stronger cytotoxicity in vitro and with nontoxicity on zebrafish development, with IC_50_ values of 33.45—62.56 μM. At a concentration of 30 µg/mL, the chloroform extract of the stems inhibited the formation of  ≥ 70% of intersegmental blood vessels and 100% of subintestinal vein blood vessels when treated zebrafish embryos, indicating the chloroform extract of *R. vesicarius* stems has apparent antitumor potential [15].

2-Methoxystypandrone (**152**) from *R*. *japonicus* exhibited antiproliferative effect on Jurkat cells and the potential to treat leukemia, by reducing the mitochondrial membrane potential and increasing the accumulation of mitochondrial reactive oxygen, as shown by flow cytometry [116]. The phenolic extract from the flower parts of *R*. *acetosa* exhibited in vitro antiproliferative effects on HaCaT cells. When increasing of the extract concentration from 25 μg/mL to 100 μg/mL, the proliferation ability on HaCaT cells gradually decreased [147].

### Antidiabetes activities

Chrysophanol (**1**) and physcion (**18**) from the roots of *R*. *crispus* showed inhibition on *α*-glucosidase, with IC_50_ values of 20.1 and 18.9 μM, respectively [180]. The alcohol extract of *R*. *acetosella* displayed stronger inhibitory activity on *α*-glucosidase (roots, IC_50_ = 12.3 μM; aerial parts, IC_50_ < 10 μM), compared to the positive control, acarbose (IC_50_ = 605 μM, *p* < 0.05), revealing *R*. *acetosella* could be developed as an antidiabetic agent [168]. Moreover, the methanolic extract of *R*. *lunaria* leaves displayed remarkable kinetic of -*α*-glucosidase activity from the concentration of 3 μM by comparison with blank control [16], and the acetone fraction of *R*. *rothschildianus* leaves showed inhibitory activity against *α*-amylase and *α*-glucosidase (IC_50_ = 19.1 ± 0.7 μM and 54.9 ± 0.3 μM, respectively) compared to acarbose (IC_50_ = 28.8, 37.1 ± 0.3 μM, respectively) [105].

The hypoglycemic effects of oral administration of ethanol extract of *R*. *obtusifolius* seeds (treatment group) were compared to the control group (rabbits with hyperglycemia). The treatment group could decrease fasting glucose levels (57.3%, *p* < 0.05), improve glucose tolerance and increase the content of liver glycogen (1.5-fold, *p* < 0.01). It also not only reduced the total cholesterol, low-density lipoprotein cholesterol levels and liver enzyme levels, but increased the high-density lipoprotein cholesterol levels. The results showed that *R*. *obtusifolius* has great potential to treat diabetes [173]. In addition, phenolic components of *R. dentatus* showed the ability to ameliorate hyperglycemia by modulating carbohydrate metabolism in the liver and oxidative stress levels and upregulating PPAR*γ* in diabetic rats [14].

### Other biological activities

The vasorelaxant antihypertensive mechanism of *R*. *acetosa* was investigated in vivo and in vitro. Intravenous injection (50 mg/kg) of the methanol extract of *R*. *acetosa* (Ra.Cr) leaves caused a mean arterial pressure (MAP) (40 mmHg) in normotensive rats with a decrease of 27.88 ± 4.55% and a MAP (70 mmHg) in hypertensive rats with a decrease of 48.40 ± 4.93%. In endothelium intact rat aortic rings precontracted with phenylephrine (1 μM), Ra.Cr induced endothelium-dependent vasorelaxation with EC_50_ = 0.32 mg/mL (0.21—0.42), while in denuded endothelial rat aortic rings, EC_50_ = 4.22 mg/mL (3.2—5.42), which was partially blocked with L-NAME (10 μM), indomethacin (1 μM) and atropine (1 μM). In isolated rabbit aortic rings precontracted with phenylephrine (1 μM) and K^+^ (80 mM), Ra.Cr induces vasorelaxation and the movement of Ca^2+^ [174].

The acetone extract of *R. japonicus* showed protective activity against myocardial apoptosis, through the regulation of oxidative stress levels in cardiomyocytes (LDH, MDA, CK, SOD) and the suppression of the expression of apoptosis proteins (caspase-3, Bax, Bcl-2) on in vitro H_2_O_2_-induced myocardial H9c2 cell apoptosis [175].

The antiplatelet activity of *R*. *acetosa* and the protective mechanism on cardiovascular system were investigated yet. The extract of *R*. *acetosa* showed inhibition of the collagen-induced platelet aggregation by modulating the phosphorylation of MAPK, PI3K/Akt, and Src family kinases and inhibited the ATP release in a dose dependent manner (25—200 μg/mL) [176]. The absorption of fexofenadine was inhibited by the ethanol extract of *R*. *acetosa* to decrease the aqueous solubility of fexofenadine [177]. The hepatoprotective effect of *R. tingitanus* was investigated by an in vivo experiment, in which the ethanol extract protected effectively the CCl_4_-damaged rats by enhancing the activity of liver antioxidant enzymes. Moreover, the extract could reduce the immobility time of mice, comparable of the positive drug, clomipramine. The results indicated that *R. tingitanus* has antidepressant-like effects [78].

Stimulating the ERK/Runx2 signaling pathway and related transcription factors could induce the differentiation of osteoblasts. Fortunately, chrysophanol (**1**), emodin (**8**) and physcion (**18**) from the aqueous extract of *R. crispus* could suppress the RANKL-induced osteoclast differentiation by suppressing the MAPK/NF-κB/NFATc1 signaling axis and increas the inhibitory factors of NFATc1 [178].

Moreover, the ethanol extract of *R. crispus* could reduce the degradation of collagen by inhibiting matrix metalloproteinase (MMP-1, MMP-8, MMP-13), indicating that *R. crispus* exhibited the antiaging function [169].

The anti-Alzheimer effect of helminthosporin (**51**) from *R. abyssinicus* was investigated in PAMPA-BBB permeability research, showing that **51** inhibited obviously AChE and BChE with IC_50_ values < 3 μM. Compound **51** could not only cross the BBB with high BBB permeability, but also bind with the peripheral anion part of the cholinesterase activity site by molecular docking [80].

It is noted, *R*. *crispus,* a traditional medicinal herb in the folk with rich retinol, ascorbic acid and *α*-tocopherol in the leaves, could be used as a complementary diet [179]. Moreover, chrysophanol (**1**) and physcion (**18**) from *R*. *crispus* roots showed obvious inhibitory activity on xanthine oxidase (IC_50_ = 36.4, 45.0 μg/mL, respectively) [180].

Inhibition of human pancreatic lipase could reduce the hydrolysis of triacylglycerol into monoacylglycerol and free fatty acids [181]. Chrysophanol (**1**) and physcion (**18**) from *R*. *nepalensis* with good inhibitory activity on pancreatic lipase (Pearson's r = 0.801 and 0.755, respectively) showed the obvious potential to treat obesity [182].

## Conclusion

The genus *Rumex* distributing widely in the world with more than 200 species has a long history of food and medicinal application in the folk. These plants with rich secondary metabolites, e.g., quinones, flavonoids, tannins, stilbenes, naphthalenes, terpenes, diterpene alkaloids, lignans and other type of components, showed various pharmacological activities, such as antimicrobial, anti-inflammatory, antiviral, renal and gastrointestinal protective effects, antioxidant, antitumor and anti-diabetes effects. Particularly, quinones as the major components in *Rumex* showed stronger antibacterial activities and exerted the potential to treat kidney disease. However, detailed phytochemical studies are needed for many *Rumex* species, in order to clarify their bioactive components. Further studies and application may focus on the antitumor, anti-diabetes, anti-microbial, hepatoprotective, cardiovascular and gastrointestinal protective effects. Moreover, the toxicity or side effects for *Rumex* plants and their chemical constituents should be evaluated, in order to make the uses of *Rumex* more safety.

## Abbreviations

AChE: Acetylcholinesterase
AGS: Adenocarcinoma gastric cell line
AR: Allergic rhinitis
BBB: Blood-brain barrier
BChE: Butyrylcholinesterase
EtOAc: Ethyl acetate
HPLC: High performance liquid chromatograph
IL: Interleukin
UHPLC-Q-TOF-MS: Ultra-high performance liquid chromatography-quadrupole time-of-flight mass spectrometry
MAPK: Mitogen-activated protein kinase
MIC: Minimum inhibitory concentration
MS: Mass Spectrometry
MTT: 3-(4,5-Dimethylthiazol-2-yl)-2,5-diphenyl tetrazolium bromide
NF-*κ*B: Nuclear factor-kappa B
QGC: Quercetin-3*-O-**β*-D-glucoside
TNF-*α*: Tumor necrosis factor-*α*
